# Dietary strategies can increase cloacal endotoxin levels and modulate the resident microbiota in broiler chickens

**DOI:** 10.1016/j.psj.2023.103312

**Published:** 2023-11-20

**Authors:** Vera Perricone, Dirkjan Schokker, Alex Bossers, Anne de Bruijn, Soumya K. Kar, Marinus F.W. te Pas, Johanna M.J. Rebel, Inge M. Wouters, Ingrid C. de Jong

**Affiliations:** ⁎ Wageningen Livestock Research, Wageningen University and Research, 6700 AH Wageningen, the Netherlands; † Wageningen Bioveterinary Research, Wageningen University and Research, 8221 RA Lelystad, the Netherlands; ‡ Institute for Risk Assessment Sciences (IRAS), Utrecht University, 3508 TD Utrecht, the Netherlands

**Keywords:** broiler, diet, endotoxin, microbiota, feed additives

## Abstract

Endotoxins released from poultry feces have been associated with impaired human health. Because endotoxins are released from gram-negative intestinal bacteria, it was hypothesized that dietary strategies may influence endotoxin excretion via modulation of gut microbiota. We therefore tested dietary strategies that could potentially reduce cloacal endotoxin levels in broiler chickens. One-day-old male Ross 308 (*N* = 1,344) broilers were housed in 48 pens (*N* = 8 pens/treatment, 28 chickens per pen) and fed 1 of 6 diets for 35 days (**d**) in a 3-phase feeding program: a basic diet (**CON**) that served as the reference diet, or basic diet supplemented with butyrate (**BUT**), inulin (**INU**), medium-chain fatty acids (**MCFA**) or Original XPC™LS (**XPC**), or a high-fiber-low-protein (**HF-LP**) diet. A significant (*P* < 0.05) increase in cloacal endotoxin concentration at d 35 was observed in BUT as compared to CON. Analysis of cloacal microbiota showed a trend (*P* < 0.07) for a higher gram-negative/gram-positive ratio and for a higher relative abundance of gram-negative bacteria at d 35 (*P* ≤ 0.08) in BUT and HF-LP as compared to CON. A significant (*P* < 0.05) increase in average daily gain (**ADG**) and improved feed conversion ratio (*P* < 0.05) were observed in MCFA during the grower phase (d 14–28), and a significant (*P* < 0.05) increase in average daily feed intake (**ADFI**) was observed in MCFA during d 0 to 28. Broilers fed HF-LP had a significantly (*P* < 0.05) higher FCR and lower ADG throughout the rearing period. No treatment effects were found on footpad dermatitis, but BUT had worst hock burn scores at d 35 (*P* < 0.01) and MCFA had worst cleanliness scores at d 21 but not at d 35 (treatment*age *P* < 0.05), while INU had better cleanliness as compared to CON at d 35 (*P* < 0.05). In conclusion, especially BUT and HF-LP were able to modulate resident microbiota and BUT also increased cloacal endotoxin levels, which was opposite to our hypothesis. The present study indicates that cloacal endotoxin release can be affected by the diet but further study is needed to find dietary treatments that can reduce cloacal endotoxin release.

## INTRODUCTION

Over the last decades, the societal concern about environmental pollution generated by livestock farms, including poultry farms, has significantly increased. Numerous epidemiological studies have demonstrated an association between working in or living near a poultry farm and adverse health effects (Freidl et al., 2017; Douglas et al., 2018). Among the air pollutants emitted by poultry farms, organic particulate matter (**PM**) represents a major component. Organic PM is derived from feces, feather and skin particles, feed, and microorganisms, along with their biological products and fragments, including endotoxins (Cambra-López et al., 2011; Zhao et al., 2014; O'Brien et al., 2016). Endotoxins, also known as lipopolysaccharides (**LPS**), are a component of the outer membrane of gram-negative bacteria, which are released in the environment upon the death/degradation of the microorganism. Endotoxins are absorbed onto the surface of coarse PM (de Rooij et al., 2017), through which they enter the respiratory system, reaching the tracheobronchial and alveolar regions, where they can induce a potent inflammatory and immune response (Behbod et al., 2013; Kumar and Adhikari, 2017). Organic dust emissions from poultry farms have been postulated to play a role in explaining adverse health associated with poultry farms (Smit et al., 2017).

Poultry farms present the highest concentration of airborne endotoxins among all livestock production systems (Cambra-Lopez et al., 2010; Basinas et al., 2012), thus representing a great concern for the respiratory health of farmers and people living in the surrounding areas. Next to the farmers and local residents, the chickens are chronically exposed to endotoxins as well, and they can suffer from similar consequences. Exposure to a high concentration of airborne endotoxins has been associated with a reduction of INF-γ production from peripheral T-cells, higher plasma cortisol levels, and a reduced proportion of peripheral B-cells in chickens (Roque et al., 2015). Lorenzoni and Wideman (2008) and van der Eijk et al. (2022) also observed pulmonary hypertension in broilers following chronic airborne exposure to LPS.

All of this shows an urgent need for effective measures to mitigate airborne endotoxins from poultry farms. Numerous systems have been developed aiming either to reduce the generation of PM or its uptake in the air or to remove PM in the air within the animal house or at the ventilation exhausts (Van der Heyden et al., 2015; Winkel et al., 2017). While their effectiveness in reducing dust can be rated from good to moderate, their efficiency in removing endotoxins is actually quite limited, making it necessary to find alternative solutions (Tymczyna et al., 2007). Moreover, these systems do not reduce the endotoxin concentrations in the broiler house.

Most airborne endotoxins are derived from chicken manure bacteria. The gut of the chickens harbors a rich variety of microorganisms, dominated by bacteria, whose composition can be modulated by means of different interventions (Clavijo and Florez, 2018; Kogut, 2019; Yadav and Jha, 2019). Among others, dietary strategies are the most successful ways to modulate the gut microbiota (Jha et al., 2019; Mahmood and Guo, 2020; Ricke et al., 2020). Therefore, it could be possible to influence the gut microbiota, yielding a decrease in gram-negative bacteria, which will in turn reduce the excretion of endotoxin concentration in the feces. This involves that dietary interventions that modulate gut microbiota should not negatively affect chicken health, welfare or productivity; if these aspects are affected, it should at least be balanced by limited endotoxin-related problems in the chickens.

The present study aimed to compare different dietary interventions with respect to their effect on the abundance of gram-negative bacteria in the gut and the excretion of endotoxins in the cloacal content of broiler chickens. Six dietary interventions were selected: a basic diet, the basic diet supplemented with 4 feed additives, and a diet with high fiber and low protein content. The basic diet was selected as a control, to depict a standard commercial diet, while the diet with high fiber content was selected based on its potential to reduce the endotoxin concentration in the litter (Rebel, unpublished). The feed additives, namely butyrate, inulin, medium-chain fatty acids and Original XPC™_LS_ (Diamond V, Cedar Rapids, IA), have been chosen according to their ability to decrease gram-negative taxa or to increase gram-positive taxa, thus to modulate the gram-negative/gram-positive ratio. Although to the best of our knowledge no one has ever directly investigated the effect of such dietary interventions on the overall intestinal gram-negative population, data in literature showed their ability to modulate the level of certain, mainly pathogenic, gram-negative bacteria, for example, by promoting competitive exclusion by providing a growth substrate for specific, gram-positive bacteria in the gut (Xu et al., 2003; Nabizadeh, 2012; Nguyen et al., 2018; Shang et al., 2018; Wu et al., 2018, 2019; Feye et al., 2019) or inhibiting growth of gram-negative bacteria (Gomez-Osorio et al., 2021). Dietary fiber can modify the gut microbiome and promote the growth of cellulolytic bacteria, predominantly gram-positive species, including the *Lactobacillus* genus and *Ruminococcaceae* family (Singh and Kim, 2021). The increased abundance of these beneficial bacteria is responsible for controlling pathogenic bacteria, primarily gram-negative bacteria, through various mechanisms, including competitive exclusion and the production of short-chain fatty acids (Yadav and Jha, 2019). We therefore hypothesized that the 5 selected diets were able to modulate the gut microbiome and reduce the gram-negative taxa, resulting in reduced fecal endotoxin secretion, as compared to the basic diet. Dietary interventions are an important factor affecting performance, excreta moisture, and litter quality, which in turn can affect animal welfare and air quality (Swiatkiewicz et al., 2017). Therefore, growth performance, litter moisture, animal behavior, and welfare parameters have also been evaluated in the present trial to determine any negative or positive side effects of the treatments. We hypothesized that performance, behavior and welfare would be comparable or positively affected as compared to the basic diet treatment group for the diets with butyrate, inulin, medium-chain fatty acids and Original XPC™_LS_. For the high fiber/low protein diet we expected a negative effect on performance (Jha and Mishra, 2021).

## MATERIALS AND METHODS

The experiment was conducted at the experimental research facility of Wageningen University and Research. All procedures complied with the Dutch law on animal experiments; the project was approved by the Central Commission on Animal Experiments (license number AVD4010020197985) and the experiment by the Ethical Committee of Wageningen University & Research, the Netherlands; experiment no. 2019.D-0009.001.

### Experimental Design, Housing, and Management

A total of 1,344 one-day-old Ross 308 male broiler chickens were obtained from a commercial hatchery (Probroed & Sloot, Groenlo, The Netherlands) and enrolled in the experimental trial that lasted 35 days (**d**). Upon arrival at the experimental facility, the birds were wing-tagged and randomly allotted to 1 of 6 dietary treatments in a completely randomized block design. The chickens were distributed over 48 floor pens (28 birds/pen) within 8 blocks of 6 pens in 2 identical climate-controlled rooms. Each pen was provided with plywood panels on each side to prevent cross-contamination between adjacent pens. The temperature at placement of the birds was set at 34°C and was gradually decreased to 20°C at 35 d of age. A continuous light program was applied during the first 3 d and was thereafter changed to 18L:6D until the end of the experiment, with a light intensity of 20 lux at bird height. Each pen measured 1.10 × 1.90 m (L × W) and was provided with wood shavings (1.0 kg/m^2^) and 1 perch (length 150 cm, height 2 cm). Feed was provided via a round feeder (diameter: 35 cm) hanging in the pen. Water was provided via 7 nipples along the side wall of a pen. Chickens were vaccinated against infectious bronchitis before arrival at the experimental facility and on d 25, and against Newcastle disease at d 15.

Feed and water were provided ad libitum. The birds were observed twice per day to monitor animal health. Mortality (number of chickens found dead in the pen) and culls (number of chickens euthanized because of compromised health or being extremely small) were recorded daily. The weight of dead chickens was recorded, and if known, also the reason for the mortality.

### Experimental Diets

The experimental diets were formulated and produced by ForFarmers, Lochem, The Netherlands. A 3-phase feeding program was applied: a starter diet was provided from d 0 to 14, a grower diet from d 14 to 28, and a finisher diet from d 28 to 35. Six experimental groups were included in the trial, as follows: control (**CON)**, which received the basic diet without any supplementation; **BUT**, which received the basic diet supplemented with sodium butyrate (0.27%, Excential Butycoat, Orffa, Werkendam, the Netherlands); **INU**, which received the basic diet supplemented with a source of inulin (0.30%; FIBROFOS60, Speerstra Feed Ingredients, Lemmer, The Netherlands); **MCFA**, which received the basic diet supplemented with a mixture of medium-chain fatty acids (**MCFA**) (0.20%; Aromabiotic, Nuscience, Belgium); **XPC**, which received the basic diet supplemented with Original XPC™_LS_ (0.12%; Diamond V, Cedar Rapids, IA); and lastly, **HF-LP**, which received a diet with higher fiber and lower protein content compared to CON. The diets were formulated to meet or exceed the requirements of broiler chickens (CVB, 2018) and to be iso-energetic within each phase. The ingredients and the calculated and analyzed nutrient composition of the diets are given in Table 1. All diets were pelleted (starter diets as 2 mm diameter pellets, grower and finisher diets as 3 mm diameter pellets).

**Table 1:** Composition (%), calculated and analyzed nutrients of the experimental diets.

Ingredients (%)	Starter (0–14 d)						Grower (14–28 d)						Finisher (28–35 d)					
	CON	BUT	INU	MCFA	XPC	HF-LP	CON	BUT	INU	MCFA	XPC	HF-LP	CON	BUT	INU	MCFA	XPC	HF-LP
Wheat	30.00	30.00	30.00	30.00	30.00	30.00	30.00	30.00	30.00	30.00	30.00	30.00	30.00	30.00	30.00	30.00	30.00	32.37
Corn	27.58	27.58	27.58	27.58	27.58	30.15	30.61	30.61	30.61	30.61	30.61	32.93	35.00	35.00	35.00	35.00	35.00	35.00
Rapeseed meal	2.00	2.00	2.00	2.00	2.00	-	2.00	2.00	2.00	2.00	2.00	-	2.78	2.78	2.78	2.78	2.78	-
Soybean meal	26.73	26.73	26.73	26.73	26.73	19.56	22.68	22.68	22.68	22.68	22.68	16.41	17.77	17.77	17.77	17.77	17.77	11.10
Sunflower seed meal	3.00	3.00	3.00	3.00	3.00	6.72	4.00	4.00	4.00	4.00	4.00	7.13	5.00	5.00	5.00	5.00	5.00	9.06
Potato protein	-	-	-	-	-	2.00	-	-	-	-	-	2.00	-	-	-	-	-	2.00
Oat hulls	1.00	1.00	1.00	1.00	1.00	2.50	1.00	1.00	1.00	1.00	1.00	2.50	1.00	1.00	1.00	1.00	1.00	2.50
Soybean oil	3.39	3.39	3.39	3.39	3.39	2.76	3.99	3.99	3.99	3.99	3.99	3.35	3.55	3.55	3.55	3.55	3.55	3.06
Palm oil	0.50	0.50	0.50	0.50	0.50	0.50	0.50	0.50	0.50	0.50	0.50	0.50	0.50	0.50	0.50	0.50	0.50	0.50
Sodium bicarbonate	0.27	0.27	0.27	0.27	0.27	0.29	0.30	0.30	0.30	0.30	0.30	0.31	0.29	0.29	0.29	0.29	0.29	0.32
Limestone	1.38	1.38	1.38	1.38	1.38	1.41	1.09	1.09	1.09	1.09	1.09	1.13	1.06	1.06	1.06	1.06	1.06	1.10
Monocalcium phosphate	0.91	0.91	0.91	0.91	0.91	0.96	0.58	0.58	0.58	0.58	0.58	0.63	0.14	0.14	0.14	0.14	0.14	0.19
Salt	0.13	0.13	0.13	0.13	0.13	0.12	0.09	0.09	0.09	0.09	0.09	0.08	0.10	0.10	0.10	0.10	0.10	0.08
L-Lysine HCl	0.32	0.32	0.32	0.32	0.32	0.33	0.34	0.34	0.34	0.34	0.34	0.33	0.35	0.35	0.35	0.35	0.35	0.36
DL-methionine	0.27	0.27	0.27	0.27	0.27	0.23	0.25	0.25	0.25	0.25	0.25	0.21	0.20	0.20	0.20	0.20	0.20	0.17
L-threonine	0.09	0.09	0.09	0.09	0.09	0.07	0.11	0.11	0.11	0.11	0.11	0.08	0.11	0.11	0.11	0.11	0.11	0.09
Valine 98	0.01	0.01	0.01	0.01	0.01	-	0.05	0.05	0.05	0.05	0.05	0.01	0.05	0.05	0.05	0.05	0.05	0.01
Axtra Phy 5000L	0.01	0.01	0.01	0.01	0.01	0.01	0.01	0.01	0.01	0.01	0.01	0.01	0.01	0.01	0.01	0.01	0.01	0.01
Premix Xylanase	0.10	0.10	0.10	0.10	0.10	0.10	0.10	0.10	0.10	0.10	0.10	0.10	0.10	0.10	0.10	0.10	0.10	0.10
Premix Maxiban	0.30	0.30	0.30	0.30	0.30	0.30	-	-	-	-	-	-	-	-	-	-	-	-
Premix Salinomycin	-	-	-	-	-	-	0.30	0.30	0.30	0.30	0.30	0.30	-	-	-	-	-	-
Premix Broiler 0–20	2.00	2.00	2.00	2.00	2.00	2.00	2.00	2.00	2.00	2.00	2.00	2.00	-	-	-	-	-	-
Premix Broiler 20–40	-	-	-	-	-	-	-	-	-	-	-	-	2.00	2.00	2.00	2.00	2.00	2.00
Butycoat	-	0.267	-	-	-	-	-	0.267	-	-	-	-	-	0.267	-	-	-	-
Fibrofos 60	-	-	0.300	-	-	-	-	-	0.300	-	-	-	-	-	0.300	-	-	-
Aromabiotic Poultry	-	-	-	0.200	-	-	-	-	-	0.200	-	-	-	-	-	0.200	-	-
Diamond V	-	-	-	-	0.125	-	-	-	-	-	0.125	-	-	-	-	-	0.125	-
Diamol	0.300	0.033	-	0.100	0.175	0.300	0.300	0.033	-	0.100	0.175	0.300	0.300	0.033	-	0.100	0.175	0.300
Calculated nutrients (g/kg)																		
AMEn broiler (kcal/kg)	2900	2900	2900	2900	2900	2900	2985	2985	2985	2985	2985	2985	3010	3010	3010	3010	3010	3010
Crude protein	210	210	210	210	210	200	197	197	197	197	197	188	182	182	182	182	182	173
Crude fat	65	65	65	65	65	59	72	72	72	72	72	66	68	68	68	68	68	63
Crude fiber	36	36	36	36	36	42	37	37	37	37	37	42	38	38	38	38	38	44
Crude ash	67	67	67	67	67	66	60	60	60	60	60	59	53	53	53	53	53	52
Starch brunt	364	364	364	364	364	381	383	383	383	383	383	399	411	411	411	411	411	426
Calcium	9.9	9.9	9.9	9.9	9.9	9.9	8.3	8.3	8.3	8.3	8.3	8.3	6.3	6.3	6.3	6.3	6.3	6.3
Phosphorus	5.8	5.8	5.8	5.8	5.8	5.8	5.0	5.0	5.0	5.0	5.0	5.0	4.0	4.0	4.0	4.0	4.0	4.0
Sodium	1.4	1.4	1.4	1.4	1.4	1.4	1.3	1.3	1.3	1.3	1.3	1.3	1.3	1.3	1.3	1.3	1.3	1.3
Chloride	2.0	2.0	2.0	2.0	2.0	2.0	1.8	1.8	1.8	1.8	1.8	1.8	1.8	1.8	1.8	1.8	1.8	1.8
6-Phytase E4a1640 (ftu)	500	500	500	500	500	500	500	500	500	500	500	500	500	500	500	500	500	500
Dig. lysine	11.5	11.5	11.5	11.5	11.5	10.9	10.8	10.8	10.8	10.8	10.8	10.3	9.9	9.9	9.9	9.9	9.9	9.4
Dig. methionine	5.5	5.5	5.5	5.5	5.5	5.2	5.1	5.1	5.1	5.1	5.1	4.9	4.6	4.6	4.6	4.6	4.6	4.4
Dig. met+cys	8.4	8.4	8.4	8.4	8.4	8.0	7.9	7.9	7.9	7.9	7.9	7.5	7.2	7.2	7.2	7.2	7.2	6.9
Dig. threonine	7.1	7.1	7.1	7.1	7.1	6.8	6.8	6.8	6.8	6.8	6.8	6.5	6.3	6.3	6.3	6.3	6.3	6.0
Dig. tryptophan	2.28	2.28	2.28	2.28	2.28	2.15	2.10	2.10	2.10	2.10	2.10	2.00	1.90	1.90	1.90	1.90	1.90	1.81
Dig. Isoleucine	7.5	7.5	7.5	7.5	7.5	7.3	7.0	7.0	7.0	7.0	7.0	6.8	6.3	6.3	6.3	6.3	6.3	6.1
Dig. Arginine	12.2	12.2	12.2	12.2	12.2	11.6	11.3	11.3	11.3	11.3	11.3	10.8	10.4	10.4	10.4	10.4	10.4	9.9
Dig. Valine	8.4	8.4	8.4	8.4	8.4	8.2	8.2	8.2	8.2	8.2	8.2	7.8	7.5	7.5	7.5	7.5	7.5	7.2
Dig. G+S	15.9	15.9	15.9	15.9	15.9	15.3	14.8	14.8	14.8	14.8	14.8	14.4	13.6	13.6	13.6	13.6	13.6	13.2
Retainable P broiler	4.4	4.4	4.4	4.4	4.4	4.4	3.7	3.7	3.7	3.7	3.7	3.7	2.8	2.8	2.8	2.8	2.8	2.8
Nicar (mg/kg)	50	50	50	50	50	50	-	-	-	-	-	-	-	-	-	-	-	-
Narasin (mg/kg)	50	50	50	50	50	50	-	-	-	-	-	-	-	-	-	-	-	-
Salinomycin (mg/kg)	-	-	-	-	-	-	70	70	70	70	70	70	-	-	-	-	-	-
Analyzed nutrients (g/kg)																		
Crude ash	66.1	64.7	64.1	65.1	65.2	64.8	59.6	57.6	55.8	50.3	56.8	48.7	51.5	50.6	49.3	50.4	51.3	51.4
Crude fiberbazin	34.7	33.9	32.3	31.7	34.1	43.4	34.5	36.7	34.6	34.8	34.2	41.4	34.9	36.7	36.7	36.8	37.3	42.9
Crude protein	209.9	208.8	209.2	210.1	210.5	199.0	197.5	195.8	195.8	197.0	193.7	188.2	180.4	180.8	179.6	179.1	180.9	170.2
Starch brunt	353.7	353.5	354.9	362.3	356.1	363.2	378.8	363.8	366.9	376.3	380.1	388.8	386.8	396.4	388.6	411.6	413.4	408.6
Crude fat	65.6	65.1	64.4	70.8	69.8	62.1	69.8	76.9	75.8	71.9	74.5	65.6	67.4	68.0	64.9	68.4	67.3	62.3

CON, control; BUT, butyrate; INU, inulin; MCFA, medium-chain fatty acids; XPC, Diamond XPC; HF-LP, high fiber-low protein. Dig = digestible.

### Technical Performance

Body weight (**BW**) and feed intake were determined on pen basis at d 0, 7, 14, 21, 28, and 35. Feed intake was measured as the difference between provided and remaining feed. Average daily gain (**ADG**), average daily feed intake (**ADFI**), and feed conversion ratio (**FCR**) were calculated for each feeding phase, that is, starter (0–14 d), grower (14–28 d), and finisher (28–35 d), as well as for the overall experimental period (0–35 d). FCR was adjusted for BW of dead or culled animals. Real mortality (chickens found dead) and total mortality (chickens found dead plus culls) were calculated over the entire experimental period (0–35 d).

### Microbiota Sampling and Processing

At d 14, 21, and 28, cloacal content was collected with sterile cotton-tipped swabs from the same 5 chickens per pen. For each time point, samples from the 5 chickens were pooled per pen for microbiota and endotoxin concentrations analysis. Only if a chicken died was it replaced by another randomly selected chicken. After collection, the swabs were immediately placed in dry ice and further stored at −80°C until analysis.

The 5 individual cloacal swabs per pen were pooled per pen in 2 mL pyrogen-free water with 0.05% Tween-20. Of this, 1 mL was used for microbiota analysis and 1 mL for endotoxin analysis. Microbial DNA was isolated from cloacal swabs according to the PureLink microbial DNA isolation kit (Thermo Fisher Scientific, Waltham, MA). Following extraction, the DNA extracts were quantified with Invitrogen Qubit 3.0 Fluorometer and stored at −20°C for further processing. The hypervariable regions V3+V4 of the 16S rRNA gene were amplified in a limited-cycles PCR with the primers CVI_V3-forw CCTACGGGAGGCAGCAG and CVI_V4-rev GGACTACHVGGGTWTCT. The following amplification conditions were used as previously described (Jurburg et al., 2019): 98°C for 2 m, followed by 20 cycles of 98°C for 10 s, 55°C for 30 s, and 72°C for 10 s, and finally by 72°C for 7 min. PCR products were checked on TapeStation (Agilent, Santa Clara, CA) and after barcoding subsequently sequenced on a MiSeq sequencer (Illumina Inc., San Diego, CA) using a version 3 paired-end 300 bp kit.

Sequence processing and statistical analyses were performed in R 4.0.2. (R Core Team, 2020). The amplicon sequences were demultiplexed and subsequently filtered, trimmed, error-corrected, dereplicated, chimera-checked, and merged using the dada2 package (v.1.16.0 (Callahan et al., 2016)). By using the standard parameters except for *TruncLength* = (270,220), *trimLeft* = (25,33), and *minOverlap* = 10, reads were classified against the SILVA v.138 database.

For this experiment, samples were filtered prior to analyses on *sample_sums* to be equal to or higher than 10,000, resulting in 1 sample being excluded from the data. For alpha-diversity-based analyses, the data were rarefied to 18,796 per sample (*rarefy_even_depth*) with *set.seed* (12345). The final and rarefied dataset contained 230 genera and 2,560 amplicon sequence variants (**ASV**), respectively. The NA percentages at ASV level over all samples were 1.26% for phylum, 8.37% for family and genus.

### Gram^−^/Gram^+^ Ratio in Cloacal Samples

In order to evaluate the effectiveness of the selected dietary interventions to modulate the intestinal gram-negative population, the bacterial groups were classified according to their gram stain. Classifying the bacterial groups was performed manually in 3 steps. Step 1, the PSORTdb was accessed on 16 November 2020 and the file “Experimental-PSORTdb-v2.00-v3.00” was downloaded. This contains 143 organisms with their respective gram stain. Step 2, this PSORTdb list was cross-referenced with our bacterial groups (genus level), the portion that was not annotated was manually curated by accessing different repositories. We have annotated 138 bacterial groups; 28 gram-negative, 84 gram-positive, 1 gram-variable, 2 inconclusive, and 23 not determined (Supplementary File S1).

### Endotoxin Analysis

The endotoxin concentration in the cloacal swabs was determined on the same samples as used for microbiota analysis. An aliquot of pooled cloacal samples in pyrogen-free water with 0.05%Tween-20 was transported to Utrecht University on dry ice. There, samples were stored at −80°C until further processing. Each pooled cloacal sample was thawed at room temperature, transferred to 15 mL polyproline tubes (Greiner Bio One bv, Etten Leur, the Netherlands), and agitated for 1 h at an end-over-end roller at room temperature. Following 15 min of centrifugation at 1,000 × *g*, the supernatant was aliquoted and stored frozen at −20°C till analysis in glass tubes, then rendered pyrogen free by being heated for 4 h at 200°C. Endotoxin content in the samples was analyzed by testing the samples in a 1:1,000 dilution with pyrogen-free water (B. Braun Medical Devices, Oss, the Netherlands) in a kinetic chromogenic limulus amebocyte lysate (**LAL**)-assay (Lonza, Verviers, Belgium), as described previously and in accordance with recommendations by Spaan et al. (2008). Endotoxin content was expressed as Endotoxin Units per mL (**EU/mL**). The mean coefficient of variation (**CV%**) for repeated LAL-analysis was 9.2% based on a random sample of 30 cloaca samples.

### Litter Dry Matter

Litter samples were collected at d 0, 7, 14, 21, 28, and 35 from each pen. The samples were collected from 5 locations per pen, thoroughly mixed, and subsequently dried to determine the dry matter content. Dry matter was gravimetrically determined by drying at a constant weight at 103°C (ISO, 6496). After sample collection at d 28, new wood shavings (2 kg) were added to each pen. The addition of new bedding was not originally planned but became necessary due to a fast deterioration of litter quality.

### Footpad Dermatitis, Hock Burn, Cleanliness, and Gait Scores

At d 21 and 35, the same 5 birds per pen selected for cloacal sampling were inspected for footpad dermatitis (**FPD**), hock burn (**HB**), and cleanliness. FPD and HB were scored on a scale from 0 (no lesions) to 4 (severe lesions on the foot or hock) (WelfareQuality, 2009). Cleanliness was scored by inspection of the belly on a scale between 0 (clean) and 3 (very dirty) (WelfareQuality, 2009). To assess the quality of locomotion, at d 35 gait score was determined on the same 5 chickens used for FPD, HB, and cleanliness scoring. Gait scoring was performed according to Kestin et al. (1992), on a scale from 0 to 5: 0: normal, dextrous, agile; 1: slight abnormality; 2: identifiable abnormality; 3: obvious abnormality, affects ability to move; 4: severe abnormality, only takes few steps; and 5: incapable of walking. All observations were performed by trained observers.

### Behavioral Observations

Direct behavioral observations were carried out at d 8, 20, and 29. Behavior was observed by scan sampling during 3 time periods, starting at 1000 h, 1230 h, and 1500 h. For each scan of 2 min per pen, the behavior of all birds in a pen was scored by counting the number of birds engaged in each activity according to the ethogram in Supplementary File S2. Behavioral observations were carried out simultaneously in the 2 rooms by 2 observers, who were trained beforehand. Each observer scored 1 room per observation interval and switched rooms between observation sessions.

### Statistical Analysis

One pen belonging to the CON group was excluded from all the analyses due to a problem in the humidification system positioned above this pen, which resulted in increased moisture of the pen that affected litter quality and technical performance. This pen was therefore considered not to be representative of the treatment. A pen within a room was considered the experimental unit, and nonsignificant effects for room were excluded in the final model. Differences of *P* < 0.05 were considered statistically significant, while 0.05 ⩽ *P* ⩽ 0.10 were considered a trend.

Microbiota statistical analyses were performed within the R environment (version 3.6.1), where we used the *phyloseq* (version 1.28.0 (McMurdie and Holmes, 2013)), *vegan* (version 2.5-7 (Oksanen et al., 2013)), *microbiome* (version 1.6.0 (Lahti and Shetty, 2017)), and *EcolUtils* (Salazar, 2020) packages to calculate alpha and beta diversity. For alpha diversity we used the command *estimate_richness*, and selected the observed species, Shannon index, and Pielou's evenness. For visualization of the beta diversity, we first performed a principal coordinate analysis (**PcoA**) with Bray-Curtis dissimilarity. Second, a permutational multivariate analysis of variance using distance matrices was performed (*adonis* and *adonis.pair*), followed by a permutation test for homogeneity of multivariate dispersions (*betadisper*).

The analyses of the endotoxin concentration, litter dry matter, performance, and behavior were performed using GenStat (Version 19.1, VSN International). The normality of the data was checked with residual plots. A natural log transformation of the aggregated measure was applied when variance was increased for increased levels of measures. Scores for FPD, HB, cleanliness, and gait were analyzed with IRCLASS followed by a residual maximum likelihood (**REML**) procedure. Age, treatment, and the interaction treatment × time were included as fixed effects. For performance parameters (BW, ADG, ADFI, FCR, mortality) and endotoxin concentration, a general ANOVA was used to test for the effect of the treatment. Scan sampling data of the behavior were analyzed by a REML procedure. Proportions of chickens showing resting behavior were calculated. Resting was the major activity. The proportion of chickens showing all other behaviors was calculated as the proportion of the chickens showing a specific behavior relative to the total number of chickens showing other behavior than resting. The analysis accounted for treatment, age, observation session, and their interactions as fixed effects. Litter dry matter of wk 1 to 4 was analyzed by REML according to a random regression model, accounting for the interaction treatment × time as a fixed effect. Litter dry matter at d 35 was analyzed separately by means of a general ANOVA, with dry matter content at d 28 as the covariate. When a treatment effect was found significant, Dunnett's test was used as a post hoc test to compare each of the experimental treatments against CON.

## RESULTS

### Cloacal Endotoxin Concentration

Cloacal endotoxin concentrations are reported in Table 2. A significant diet effect on cloacal endotoxin concentrations was observed at d 35 (*P* = 0.024), while no treatment effect on endotoxin concentrations was found at d 21, and a trend (*P* = 0.077) on d 14. Dunnett's test revealed no differences between the experimental diets and CON at d 14, while at d 35 BUT had higher cloacal endotoxin concentration compared to CON (Δ = 255.5 EU/mL; *P* < 0.05).

**Table 2:** Average endotoxin concentration in cloacal content of broiler chickens.

Endotoxin concentration	Age	CON	BUT	INU	MCFA	XPC	HF-LP	SED	*P*_diet_
Log-transformed	d 14	5.438	5.269	5.393	5.219	5.417	5.981	0.2605	0.077
	d 21	5.355	5.049	5.16	5.023	5.276	5.694	0.3429	0.406
	d 35	5.153	6.157*	5.299	5.851	5.451	5.96	0.3279	**0.024**
Back-transformed means (EU/mL)	D 14	249.0	212.8	249.6	213.7	246.9	442.5	67.9	
	D 21	237.1	170.2	249.2	175.0	252.7	395.2	93.6	
	D 35	203.2	458.7	245.3	414.7	284.9	425.9	110.2	

Boldface indicates a significant effect (*P* < 0.05).

⁎ Values are significantly different (*P* < 0.05) compared to CON (Dunnett's test). Values are expressed as natural log _(EU/mL)_ and SED (upper part of the table) and as back-transformed means and SED (lower part of the table). CON, control; BUT, butyrate; INU, inulin; MCFA, medium-chain fatty acids; XPC, Diamond XPC; HF-LP, high fiber-low protein; SED, standard error of differences. As statistical analysis was performed on the log-transformed values; *P* values are only presented in this part of the table.

### Microbiota Composition and Gram-Negative/Gram-Positive Ratio

Only trends were observed in the alpha-diversity measures, that is, observed species, Shannon index, and Pielou's evenness, at d 35 when comparing the treatment groups to the control group. No treatment effects were seen at all at d 14 and d 21 (Table 3). In the Observed species at d 35, a trend for higher diversity was observed when comparing BUT, MCFA, and HF-LP to the CON group (*P* = 0.07, *P* = 0.07, and *P* = 0.06, respectively) (Table 3). Similarly, for the Shannon index and evenness, these 3 groups tended to have a higher diversity at d 35 (BUT vs. CON (*P* = 0.06), MCFA vs. CON (*P* = 0.06), and HF-LP vs. CON (*P* = 0.05) for Shannon index, and BUT vs. CON (*P* = 0.05), MCFA vs. CON (*P* = 0.07) and HF-LP vs. CON (*P* = 0.05) for Pielou's evenness) (Table 3).

**Table 3:** Alpha-diversity indices of the fecal microbiota (Observed species, Pielou's evenness and Shannon index) for the treatments at d 14, 21, and 35 of age, including the *P* value when comparing the treatments to the control (CON) group.

		Observed species	Observed species	*T* test	Pielou's evenness	Pielou's evenness	*T* test	Shannon	Shannon	*T* test
Day	Treatment	Mean	SD	*P* value	Mean	SD	*P* value	Mean	SD	*P* value
14	CON	171	60	-	0.51	0.16	-	2.67	0.99	-
14	BUT	217	54	0.6	0.62	0.09	0.68	3.35	0.55	0.76
14	INU	202	25	0.86	0.46	0.09	1.00	2.50	0.54	1.00
14	MCFA	166	52	0.96	0.45	0.05	1.00	2.31	0.34	1.00
14	XPC	193	56	0.94	0.48	0.12	1.00	2.58	0.75	1.00
14	HF-LP	206	52	0.86	0.62	0.13	0.77	3.32	0.86	0.76
21	CON	270	57	-	0.60	0.12	-	3.38	0.80	-
21	BUT	278	59	1.00	0.59	0.12	1.00	3.34	0.77	1.00
21	INU	258	89	1.00	0.58	0.17	1.00	3.25	1.09	1.00
21	MCFA	241	37	1.00	0.55	0.04	1.00	3.07	0.28	1.00
21	XPC	225	87	1.00	0.53	0.10	1.00	2.90	0.77	0.95
21	HF-LP	283	55	1.00	0.62	0.09	1.00	3.58	0.62	1.00
35	CON	219	103	-	0.57	0.11	-	3.10	0.81	-
35	BUT	343	51	0.07	0.71	0.07	0.05	4.21	0.45	0.06
35	INU	250	75	0.53	0.54	0.10	0.58	3.03	0.69	1.00
35	MCFA	346	82	0.07	0.69	0.06	0.07	4.08	0.51	0.06
35	XPC	295	97	0.32	0.65	0.10	0.35	3.72	0.78	0.30
35	HF-LP	352	50	0.06	0.72	0.08	0.05	4.28	0.54	0.05

BUT, butyrate; INU, inulin; MCFA, medium-chain fatty acids; XPC, Diamond XPC; HF-LP, high fiber-low protein.

**Figure 1:**
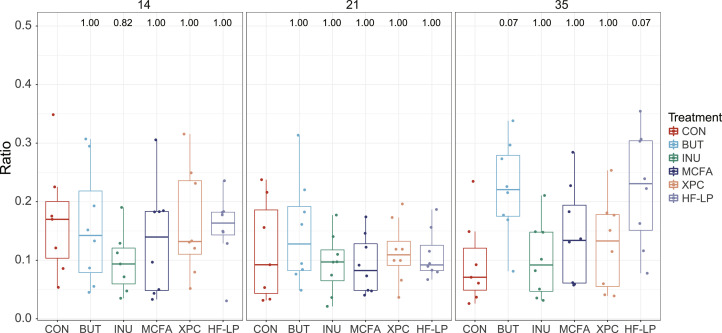
Gram−/gram+ ratio for at d 14 (left), 21 (middle), and 35 (right) of age. CON, control; BUT, butyrate; INU, inulin; MCFA, medium-chain fatty acids; XPC, Diamond XPC; HF-LP, high fiber-low protein. Significance levels of diets as compared to CON are shown above the bars in the graphs.

**Figure 2:**
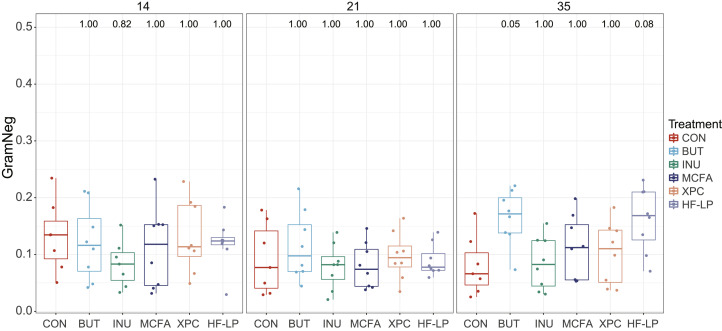
Relative abundance of gram-negative stained bacteria for all groups at d 14 (left), 21 (middle), and 35 (right) of age. CON, control; BUT, butyrate; INU, inulin; MCFA, medium-chain fatty acids; XPC, Diamond XPC; HF-LP, high fiber-low protein. Significance levels of diets as compared to CON are shown above the bars in the graphs.

We specifically focused on the gram-negative/gram-positive (**gram^−^/gram^+^**) ratio and the gram-negative relative abundance, because of their involvement in the production of endotoxins. As observed for the alpha diversity measures, we only observed some trends at d 35. A trend for a higher gram−/gram+ ratio at d 35 was observed for BUT compared with CON (*P* = 0.07) and HF-LP compared with CON (*P* = 0.07) (Figure 1). Similarly, BUT and HF-LP tended to have a higher relative abundance of gram-negative bacteria compared to CON (*P* = 0.05 and *P* = 0.08, respectively) but no treatment effects were found at d 14 and 21 (Figure 2).

**Figure 3:**
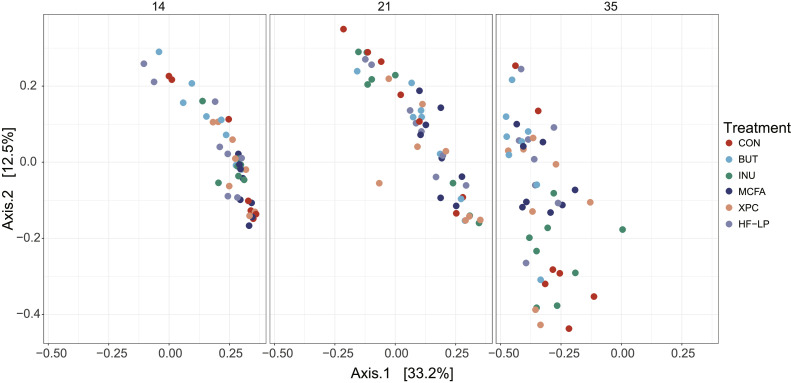
Principal coordinate analysis using the Bray-Curtis dissimilarities of all samples, for d 14 (left), d 21 (middle), and d 35 (right) of age. Each symbol represents a sample. The first axis explains 33.2% of the variation and the second axis represents 12.5% of the variation. CON, control; BUT, butyrate; INU, inulin; MCFA, medium-chain fatty acids; XPC, Diamond XPC; HF-LP, high fiber-low protein.

To assess the beta diversity, that is, the differences between the day of sampling and the different treatments compared to the control, we first performed a Principal Coordinate Analysis (Figure 3), which shows a clear turnover of the microbiota composition in time, where the first axis (age) explains 33.2% of the variation and the second axis 12.5% of the variance. This change was also observed when performing a permutational multivariate analysis of variance using dissimilarities (i.e., assessing the beta-diversity) on the microbiota data, resulting in a significant effect for the main effects age (*P* = 0.001) and treatment (*P* = 0.017) (Supplementary File S3). The permutation test for homogeneity of multivariate dispersions was significant (*P* = 0.002), however, when further testing was performed by pairwise comparisons between one of the treatments vs. the CON group per day, no significant differences were found. Thereafter, we performed a pairwise analysis for the multivariate analysis of variance using dissimilarities, but this did also not result in significant differences when comparing any of the treatments with the CON group on d 14, 21, or 35.

Stacked bar plots representing the average relative abundance per treatment per day for 2 taxonomic levels, that is, phylum and genus are shown in Supplementary File S4 to gain more insight into the microbiota composition per treatment. For the phylum level, a clear dominance of *Firmicutes* was observed, that is, 88.2% (min = 84.5%, max = 93.6%), independent of day and treatment. When focusing on the turn-over of the microbiota composition, the *Actinobacteria* showed a slight increase on average from d 14 to 21, whereas *Bacteroidota* increased on average from d 21 to 35. Moreover, we also observed a decrease in the *Proteobacteria* from d 14 to 21 and d 21 to 35.

For the genus level we focused on the top 10 genera over day and treatment (Supplementary File S4). The highest average relative abundance, that is, 40.6% (min = 23.1%, max = 55.1%) independent of treatment and day observed was *Lactobacillus*. Furthermore, we observed some genera that fluctuated over time. *Enterococcus* showed a decrease over time, from an average of 9.6% on d 14 to 0.2% on d 35. Contrary, *Faecalibacterium* and *Streptococcus* showed an increase in average abundance over time. Where *Faecalibacterium* had a relative average abundance of 0.5% on d 14, 8.9% on d 21, and 8.8% on d 35. *Streptococcus* had a relative average abundance of 0.02% on d 14, 0.2% on d 21, and 8.9% on d 35. No significant differences were observed in the average relative abundances between treatments and the CON group at different taxonomic levels.

### Behavioral Observations

Table 4 shows the predicted means for the proportion of birds resting. No treatment effects were found, but a significant age effect was observed, with resting increasing with age (*P* < 0.001). A significant interaction between age and observation session was also observed (*P* < 0.005). Within the same day, differences were observed only at d 20, with animals resting more at 1230 h and 1500 h compared to 1000 h. Between observation days, at 1000 h, birds were resting more at d 29 compared to d 8 and d 20; at 1230 h, birds were resting more at d 20 and d 29, compared to d 8; at 1500 h, birds were resting more at d 29 compared to d 8 (Table 4). Table 5 shows the predicted means for all behaviors except resting. A significant effect of the treatment was observed for standing, with highest proportion of chickens standing in BUT (*P* = 0.011), but Dunnett's test revealed no differences between the experimental groups and CON. A trend for a treatment effect was observed for drinking behavior (*P* = 0.061), with birds in the MCFA group drinking less compared to CON. A significant age effect was found for drinking, locomotion, and foraging. Locomotion was reduced at d 29 compared to d 8 and d 20. Drinking was reduced at d 29 compared to d 20, and foraging behavior was decreased at d 20 and 29 compared to d 8 (Table 5).

**Table 4:** Predicted means (on a log scale) of the proportion of broilers resting at d 8, 20, and 29 of age and different times of the day (1000, 1230, and 1500 h), averaged over all treatments, and the *P* value for treatment (T), age (A), and per observation session (S) and their interaction.

Age	1000 h	1230 h	1500 h	SED	*P* value						
					T	A	S	TxA	TxS	AxS	TxAxS
D 8	4.217 ab	4.167 ^a^	4.245 ab	0.0525	0.544	**<0.001**	0.093	0.720	0.696	**0.005**	0.359
D 20	4.185 a	4.405 c	4.319 bc								
D 29	4.396 c	4.412 c	4.373 c								

Boldface indicates a significant effect (*P* < 0.05).

a,b,c Rows and columns with different superscript are significantly different (*P* < 0.05).

**Table 5:** Predicted means (on logit scale) per behavioral category, for different treatments (T), age (A), and per observation session (S).

	Treatment						Age (d)			Session (h)			T		A		S	
Behavior	CON	BUT	INU	MCFA	XPC	HF-LP	8	20	29	1000	1230	1500	SED	*P*	SED	*P*	SED	*P*
Eating	1.630	1.321	1.245	1.249	1.310	1.331	1.311	1.485	1.248	1.457	1.178	1.408	0.388	0.938	0.404	0.832	0.272	0.562
Drinking	1.697	0.990	1.496	0.774	1.491	1.193	1.264	1.556	1.001	1.446	1.128	1.246	0.333	0.061	0.225	**0.043**	0.235	0.377
Locomotion	0.500	0.943	0.737	0.411	0.846	0.937	1.385	0.848	−0.045	0.945	0.651	0.590	0.276	0.272	0.382	**0.014**	0.382	0.619
Standing	0.440	0.673	0.291	−0.212	0.616	0.387	0.336	0.715	0.047	0.749	0.365	−0.016	0.255	**0.011**	0.317	0.180	0.372	0.268
Preening	1.104	0.980	0.968	1.330	1.135	0.724	1.125	0.893	1.103	0.942	0.969	1.210	0.350	0.635	0.245	0.619	0.347	0.721
Stretching	0.912	0.786	1.043	0.521	0.503	0.637	0.604	0.426	1.171	0.505	0.730	0.966	0.322	0.478	0.500	0.329	0.510	0.673
Foraging	−0.101	−0.078	0.065	0.150	0.154	0.064	1.065	−0.292	−0.645	0.204	−0.009	−0.068	0.217	0.778	0.208	**<0.001**	0.208	0.419

Boldface indicates a significant effect (*P* < 0.05).

CON, control; BUT, butyrate; INU, inulin; MCFA, medium-chain fatty acids; XPC, Diamond XPC; HF-LP, high fiber-low protein; SED, standard error of differences. Note that these behaviors were calculated as proportion of chickens showing a behavior relative to the number of chickens showing other behaviors than resting.

### Footpad Dermatitis, Hock Burn, Cleanliness, and Gait

Predicted means for FPD, HB, cleanliness, and gait scores are provided in Table 6, and Supplementary File S5 shows the distribution of scores for all treatment groups. A significant effect of age was found for FPD, HB, and cleanliness (*P* < 0.001), with scores increasing (i.e., getting worse) from d 21 to d 35. A treatment effect was found for HB (*P* = 0.004) with BUT having the worst scores, but Dunnett's test revealed no differences when comparing the treatment groups to CON. No treatment effect was observed for FPD and cleanliness. The interaction treatment × age was found to be significant for cleanliness in the MCFA group (*P* = 0.035), with MCFA having the highest (worst) scores at d 21 of age. According to Dunnett's test, at d 35 INU group had a lower (better) cleanliness score compared to CON (Table 6; *P* < 0.05).

**Table 6:** Predicted means for footpad dermatitis (FPD), hock burn (HB), cleanliness at d 21 and 35 of age and *P* values for treatment, age and their interaction effect, and predicted means for gait score at d 35 of age with *P* value for treatment effect.

Indicator	Day	CON	BUT	INU	MCFA	XPC	HF-LP	Treatment		Age		Treatment × age	
								SED^1^	*P* value	SED	*P* value	SED	*P* value
FPD	21	−1.936	−2.022	−2.009	−2.441	−2.298	−2.193	0.502	0.581	0.852	**<0.001**	0.860	0.971
	35	2.170	2.258	1.947	1.399	2.203	2.208						
HB	21	−12.142	−1.374	−1.994	−2.246	−2.974	−3.657	3.211	**0.004**	2.943	**<0.001**	3.592	0.773
	35	2.744	3.365	2.943	2.009	2.658	1.631						
Cleanliness	21	0.216	0.160	0.325	0.887	0.691	0.632	0.399	0.552	0.227	**<0.001**	0.511	**0.035**
	35	3.703	3.558	2.334	2.861	2.870	3.522						
Gait	35	3.337	3.911	2.947	3.229	4.565	3.227	0.704	0.144	-	-	-	-

Boldface indicates a significant effect (*P* < 0.05).

CON, control; BUT, butyrate; INU, inulin; MCFA, medium-chain fatty acids; XPC, Diamond XPC; HF-LP, high fiber-low protein; SED, standard error of differences.

### Litter Dry Matter

The estimates of the linear regression slope of litter dry matter (wk 1–4) and dry matter at d 35 are provided in Supplementary File S6. The random regression model showed a different rate of decline of litter dry matter over time between treatments (*P* < 0.001), with the steepest slopes for BUT and CON. However, according to Dunnett's test, none of the experimental treatments differed from CON. Similarly, at d 35, a significant treatment effect was observed (*P* = 0.013) with the lowest dry matter percentage for BUT, but no differences were reported when comparing the experimental treatments with CON using Dunnett's test.

### Technical Performance

Technical performance parameters are presented in Table 7. In the starter phase (0–14 d), no treatment effect was observed for ADG and ADFI, while a significant difference was reported for FCR (*P* < 0.001). According to Dunnett's test, the FCR of broilers fed the HF-LP diet was significantly higher compared to CON (Δ = 0.035). In the grower phase (14–28 d), a significant treatment effect was observed for all the performance parameters. According to Dunnett's test, broilers in the MCFA group had higher ADG and better FCR compared to CON (Δ = 6.51 g and Δ = −0.045, respectively). Like in the starter phase, HF-LP had higher FCR compared to CON (Δ = 0.052). Even though ADFI was affected by the treatment, none of the experimental groups differed from CON using Dunnett's test. In the finisher phase (28–35 d), a treatment effect was reported for ADFI (*P* = 0.047) and a trend for ADG (*P* = 0.092), but no differences were detected when comparing the treatment groups vs. CON. Over the entire experimental period (0–35 d), all the performance parameters were affected by treatment. Compared to CON, the MCFA group had higher ADG and ADFI (Δ = 4.26 g and Δ = 4.17 g, respectively), while HF-LP had higher FCR (Δ = 0.046). Real and total mortality (mortality and culls) were not affected by treatment.

**Table 7:** Average daily gain (ADG), average daily feed intake (ADFI), feed conversion ratio (FCR), and mortality of broiler chickens per feeding phase and for the whole rearing period.

Indicator	CON	BUT	INU	MCFA	XPC	HF-LP	SED	*P* value
ADG (g)								
0–14 d	35.08	35.33	35.09	36.19	35.80	34.94	0.516	0.137
14–28 d	93.26	94.90	94.46	99.77*	97.01	89.27	1.872	**<0.001**
28–35 d	113.03	109.93	110.92	119.08	116.73	109.94	3.761	0.092
0–35 d	73.94	74.08	74.00	78.20*	76.47	71.67	1.422	**0.001**
ADFI (g)								
0–14 d	39.50	40.17	39.79	40.49	40.43	40.53	0.589	0.417
14–28 d	129.29	130.51	129.63	134.08	132.83	128.52	1.815	**0.028**
28–35 d	174.97	176.25	173.44	184.22	182.94	175.03	4.072	**0.047**
0–35 d	102.50	103.52	102.46	106.67*	105.89	102.63	1.551	**0.028**
FCR (g:g)								
0–14 d	1.125	1.137	1.134	1.119	1.130	1.160*	0.008	**<0.001**
14–28 d	1.389	1.376	1.372	1.344*	1.370	1.441*	0.015	**<0.001**
28–35 d	1.551	1.608	1.569	1.548	1.569	1.593	0.026	0.192
0–35 d	1.387	1.398	1.385	1.364	1.385	1.433*	0.011	**<0.001**
Real mortality (%)^1^	3.2	4.0	1.7	2.3	4.0	3.4	0.605	0.716
Total mortality (%)^2^	5.2	7.4	5.7	2.8	5.7	10.8	0.494	0.146

Boldface indicates a significant effect (*P* < 0.05).

⁎ Values are significantly different (*P* < 0.05) compared to CON (Dunnett's test).

1 Real mortality = animals found dead in the pen. Calculated overall the entire experimental period (0–35 d).

2 Total mortality = real mortality plus culls. Calculated overall the entire experimental period (0–35 d). CON, control; BUT, butyrate; INU, inulin; MCFA, medium-chain fatty acids; XPC, Diamond XPC; HF-LP, high fiber-low protein; SED, standard error of differences.

## DISCUSSION

Dietary strategies can potentially be used to reduce the load of gram-negative bacteria in the intestinal tract, which can in turn reduce the cloacal endotoxin release. In the present trial, 5 experimental diets were selected for their ability to directly or indirectly modulate the relative abundance of gram-negative taxa as compared to the standard commercial diet (Xu et al., 2003; Nabizadeh, 2012; Nguyen et al., 2018; Shang et al., 2018; Wu et al., 2018, 2019; Feye et al., 2019; Gomez-Osorio et al., 2021; Rebel, unpublished), and cloacal microbiota composition and endotoxin concentration was measured.

We observed that the experimental diets could indeed modulate the diversity of the microbiota, particularly at d 35, where MCFA, BUT and HF-LP showed trends of having higher observed species (richness) and Shannon index compared to the control group. In addition, as compared to the control diet, BUT and HF-LP also showed a trend of a higher gram−/gram+ ratio, but this was not observed for the MCFA treatment. It is well known that dietary fiber and protein concentration both affect the microbiota composition and/or diversity (Pan and Yu, 2014; Makki et al., 2018; Yadav and Jha, 2019; Tejeda and Kim, 2021). Dietary fiber can promote the growth of gram-positive bacteria and in this way control the abundance of primarily gram-negative bacteria through various mechanisms, including competitive exclusion and production of short-chain fatty acids (Yadav and Jha, 2019; Singh and Kim, 2021). However, in the present trial HF-LP tended to increase the cloacal endotoxin concentration, which was thus opposite to the expected and desired effect based on a preliminary pilot study. Furthermore, although not significant, the effect on gram-negative bacteria was numerically higher in HF-LP birds as compared to the other groups, which was also in contrast to expectations based on the earlier pilot study (Rebel, unpublished). It should be noted that in the previous pilot, endotoxins concentrations were determined in the feces, while in this study we used cloacal swabs, which may have caused the different results as in fecal samples microbial processes or environmental contamination could have affected the endotoxin concentration. Although inulin is also dietary fiber, here we did not find any indication of inulin-induced cloacal microbiota composition changes. This suggests that the type and dose of fructan-based fibers in combination with the basic diet as used in the present trial are not able to modulate the fecal microbiome.

BUT has been suggested to stimulate abundance of gram-positive bacteria, such as *Lagnospiraceae* or *Ruminococcaceae*, while decreasing the abundance of gram-negative bacterial, such as the *Enterobacteriaceae* family (Wu et al., 2018). However, BUT increased gram-negative bacteria abundance in the cloacal microbiota and the endotoxin excretion in the same cloacal samples in the present trial, suggesting that BUT might be a growth stimulator for gram-negative bacteria. This was, however, an unwanted effect. This shows that the butyrate we added to the basic diet cannot be considered as a suitable feed additive to reduce the cloacal endotoxin content in broiler chickens. Thus, although the diets tested in this study were not able to modulate the cloacal endotoxin concentration, or even resulted in an increase, this study showed that additives or diet composition can change the gram−/gram+ ratio in the cloacal microbiota and the cloacal endotoxin concentration. It is therefore suggested to further investigate additive concentrations and diets for the desired effect, that is, a reduced endotoxin release.

If the diets that have been applied in the present trial can modulate the cloacal microbial composition and endotoxin concentration, it is important to determine whether these diets have negative side effects on broiler welfare and performance. Overall, very few effects on behavior and welfare indicators were found for all dietary treatments, indicating that the applied additives or the HF-LP diet did not impact broiler welfare as compared to the control diet. Only a few significant treatment effects were observed. BUT resulted in worse scores for hock burn and showed the lowest values for litter dry matter content, which may be related (de Jong et al., 2014), although no effects were found for footpad dermatitis, which is usually also increased with lower litter dry matter content (de Jong et al., 2014). The more wet litter in BUT pens may also explain the higher proportion of broilers showing standing in BUT, as the wetter litter might have caused (thermal) discomfort for sitting chickens. The INU treatment resulted in a better cleanliness score as compared to CON at d 35, but INU did not result in better scores for the other welfare indicators nor resulted in dryer litter. The tendency for less drinking behavior observed in the MCFA treatment as compared to the other treatments did not have any obvious relationship with welfare scores, although MCFA had somewhat better scores for contact dermatitis than the other treatments but had worse cleanliness scores. Overall, effects on behavior and welfare were small and only found for few parameters, and no treatment showed a good welfare score, which might be due to the relatively small pens and quickly deteriorating litter quality in the pens.

Butyrate, inulin, and XPC did not significantly affect any of the measured performance traits. This was unexpected because all 3 have been described as performance-enhancing additives for broiler chickens (Nabizadeh, 2012; Abdelqader et al., 2013; Ahsan et al., 2016; Goda et al., 2018; Elnesr et al., 2020). The reasons that we did not find a positive effect on the performance could be due to the fact that the experiment was performed in our facility where no additional health challenges occurred due to the relatively high level of biosecurity, and the usually better performance under these small-scale conditions as compared to commercial conditions. Alternatively, it may be that too low concentrations of the additives were provided. However, none of the additives exhibited a nonsignificant tendency toward performance improvement, or in another direction. In addition, BUT, for example, was reported to be effective in concentrations as low as 100 mM (Scheppach et al., 1992). Furthermore, 0.63 g/kg coated BUT was effective in poultry in reducing Salmonella in the gut (Van Immerseel et al., 2005). Thus, it seems unlikely that insufficient dietary concentrations could be the reason for the ineffectiveness.

The HF-LP diet increased the FCR during the growth of the broilers, but did not affect other performance traits. The lower protein content of the diet may reduce growth traits (Alleman and Leclercq, 1997). This was indeed observed, although not significant. The MCFA diet improved the growth rate of the broilers, but at higher feed intake and with minimal effects of FCR, thus, did also not have a positive effect on performance. Thus, the applied treatments generally neither improved nor impaired performance and welfare as compared to the control diet.

In the present study, we investigated the microbial composition in cloacal swabs of chickens and its correlation with endotoxin secretion. Our particular focus was on the relationship between these 2 factors. However, a more comprehensive understanding of the effects of the different additives and HF-LP diet on gut microbiota and their mechanism of action would have been possible if we had collected samples from various gut sections. It is important to consider that the cecum is the main site of fermentation in the intestine and has the highest bacterial biodiversity (Clavijo and Florez, 2018). Analyzing the changes in the cecal microbiota composition could provide insight into whether or not the additives have an impact on physiological processes, such as fiber degradation. Previous studies have shown that there is a positive correlation between the abundance of specific taxa in cloacal and cecal communities. Therefore, cloacal samples can be a reliable proxy to evaluate the cecal microbial community (Andreani et al., 2020). In addition, by assessing the microbial composition of cloacal samples, we were able to track the evolution of the microbiome of the same animal over time, thus avoiding any potential bias caused by individual variability (Bindari and Gerber, 2022).

## CONCLUSIONS

The aim of the present study was to determine whether feed additives or an adjusted feed composition with high fiber/low protein concentration can reduce cloacal endotoxin content in broiler chickens. It is clear that the investigated feed additives (BUT, MCFA, INU, and XPC) or the alternative diet (HF-LP) were not able to generate a significant effect in the intended direction compared to the respective control diet, although we also showed that BUT and HF-LP were able to modulate the cloacal microbiota and increase the cloacal endotoxin level, without having adverse effects on broiler welfare and performance. Further research is needed to ascertain whether any other diet or additive can reduce the cloacal endotoxin concentration as compared to the standard commercial diet.

## Appendix Supplementary materials

Image, application 1

Image, application 2

Image, application 3

Image, application 4

Image, application 5

Image, application 6

## Footnotes

Supplementary material associated with this article can be found in the online version at doi:10.1016/j.psj.2023.103312.
