# Supplementary material for: Dietary strategies can increase cloacal endotoxin levels and modulate the resident microbiota in broiler chickens
Source: Poult Sci. 2023 Nov 20;103(2):103312. doi: 10.1016/j.psj.2023.103312 (PMC10762469; doi:10.1016/j.psj.2023.103312)
Supplement: Supplementary file 2 [file mmc2.docx]

**Supplementary File S2.**

Table: ethogram of the behavioral measurements

| **Behavior** | **Description** |
| --- | --- |
| Eating | Having the head in the feeder or pecking at the feeder |
| Drinking | Pecking at the drinking nipples |
| Walking | Walking, running, jumping without performing any other type of behaviour |
| Resting | Sitting or lying while not engaged in any other activities |
| Standing | Standing while not engaged in any other activities |
| Dust bath | Rubbing the head or body against the floor, lying on the side and pecking or scratching, spreading litter over the body, or shaking off the body |
| Preening | Manipulating own feathers with the beak |
| Stretching | Flapping wings, stretching, shaking feathers (not while dust bathing) |
| Sitting and ground pecking | Sit and peck at the litter at the same time |
| Foraging | Pecking and/or scratching at the litter while standing |
| Aggressive behavior | All elements of aggressive behaviour, such as hopping oriented towards another chicken, threatening, leaping, kicking, wing flapping or aggressive pecking |
| Feather pecking | Pecking at the feathers of another chick |
| Exploring | Pecking at objects, including parts of the pen, feeders or drinkers apart from the feed itself or the nipple |
| Other | Everything not defined above |
