# Supplementary material for: Dietary strategies can increase cloacal endotoxin levels and modulate the resident microbiota in broiler chickens
Source: Poult Sci. 2023 Nov 20;103(2):103312. doi: 10.1016/j.psj.2023.103312 (PMC10762469; doi:10.1016/j.psj.2023.103312)
Supplement: Supplementary file 3 [file mmc3.docx]

**Supplementary File S3.**

Table. Results of permutational multivariate analysis of variance using distance matrices when testing for *age* and *treatment.*

|  | **Df^1^** | **Sums Of Squares** | **Mean Squares** | **F Model** | **R^2^** | **Pr(>F)^2^** |
| --- | --- | --- | --- | --- | --- | --- |
| *age* | 1 | 8.718 | 8.7177 | 54.208 | 0.275 | **0.001** |
| *treatment* | 5 | 1.316 | 0.2633 | 1.637 | 0.042 | **0.017** |
| *age* * *treatment* | 5 | 1.041 | 0.2082 | 1.294 | 0.033 | 0.125 |
| Residuals | 128 | 20.585 | 0.1608 |  | 0.650 |  |
| Total | 139 | 31.660 |  |  | 1.000 |  |

^1^Degrees of freedom
