# Supplementary material for: Dietary strategies can increase cloacal endotoxin levels and modulate the resident microbiota in broiler chickens
Source: Poult Sci. 2023 Nov 20;103(2):103312. doi: 10.1016/j.psj.2023.103312 (PMC10762469; doi:10.1016/j.psj.2023.103312)

**Supplementary File S4.**

**Figure:** Compositional data on Phylum level of all groups at d14 (left), 21 (middle), and 35 (right) of age. The x-axis depicts the treatment/groups per day of age, where the left panel shows day 14, the middle day 21, and the right day 35. The y-axis depicts the relative abundance. Each color represents a different taxa; light blue, Firmicutes; blue, Actinobacteria, light green, Proteobacteria; green, Bacteroideta; pink, Other. CON, control; BUT, butyrate; INU, inulin; MCFA, medium-chain fatty acids; XPC, Diamond XPC; HF-LP, high fiber-low protein.

**
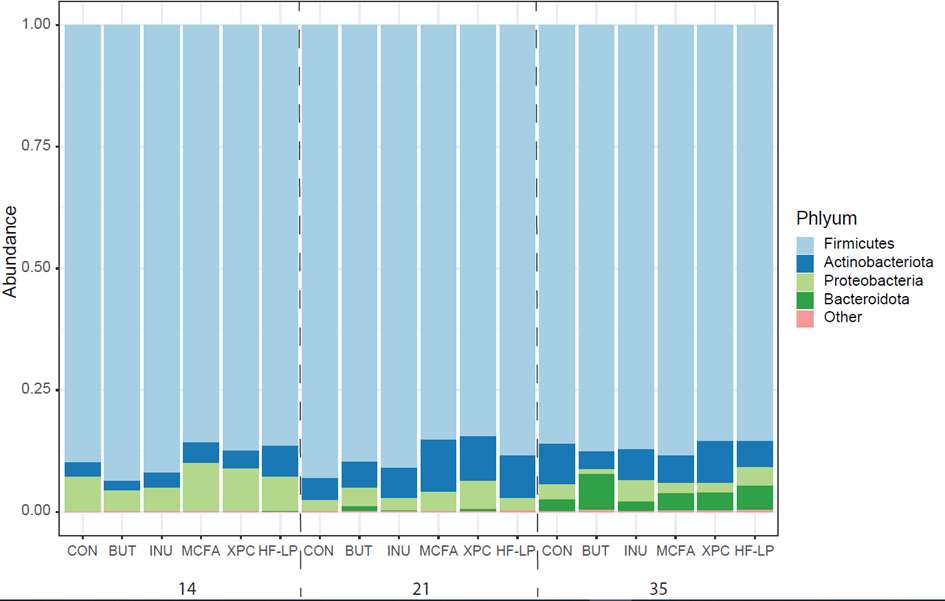
**

**Figure:** Compositional data at genus level of all groups at d14 (left), 21 (middle), and 35 (right) of age. The x-axis depicts the treatment/groups per day of age, where the left panel shows day 14, the middle day 21, and the right day 35. The y-axis depicts the relative abundance. Each color represents a different taxa; Each color represents a different taxa; light blue, [family] Lachnospiraceae; blue, Blautia, light green, Corynebacterium; green, Enterococcus; pink, Escherichia/Shigella; red, Faecalibacterium; light orange, Lactobacillus; orange, Romboutsia; lilac, Streptococcus; dark purple, Subdoligranulum; yellow, other. CON, control; BUT, butyrate; INU, inulin; MCFA, medium-chain fatty acids; XPC, Diamond XPC; HF-LP, high fiber-low protein.


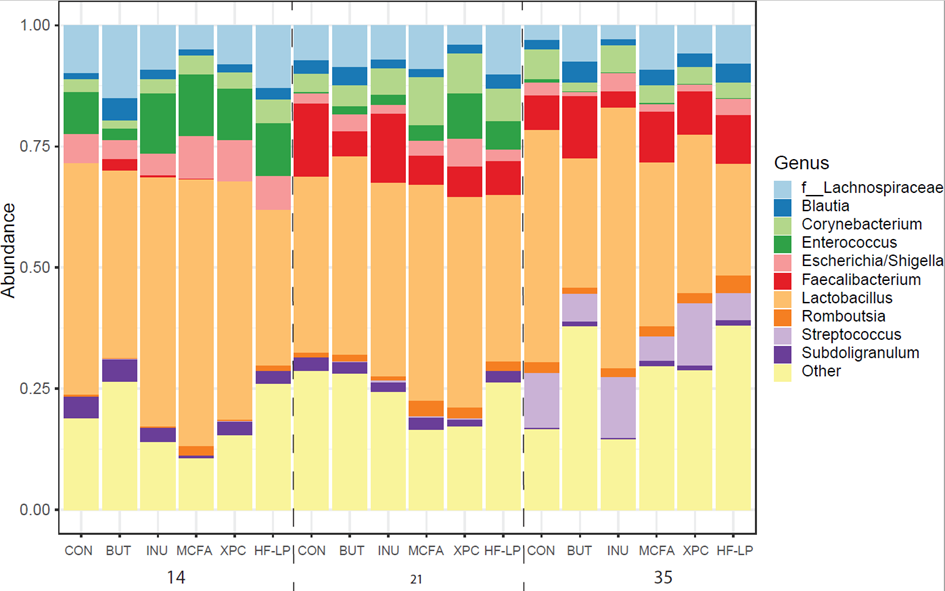

Supplement: Supplementary file 4 [file mmc4.docx]
