# Supplementary material for: Dietary strategies can increase cloacal endotoxin levels and modulate the resident microbiota in broiler chickens
Source: Poult Sci. 2023 Nov 20;103(2):103312. doi: 10.1016/j.psj.2023.103312 (PMC10762469; doi:10.1016/j.psj.2023.103312)

**Supplementary File S5.**

**Figure:.** Distribution of footpad dermatitis, hock burn, cleanliness and gait score (figures from top to bottom) at days 21 and 35 (gait score only at d35), illustrating the percentage of birds within each category for each experimental group. Footpad dermatitis and hock burn were scored on a scale from 0 (no lesions) to 4 (large, severe lesions). Cleanliness was scored on a scale from 0 (clean) to 3 (very dirty). Gait was scored on a scale from 0 (perfect) to 5 (unable to walk). CON, control; BUT, butyrate; INU, inulin; MCFA, medium-chain fatty acids; XPC, Diamond XPC; HF-LP, high fiber-low protein.


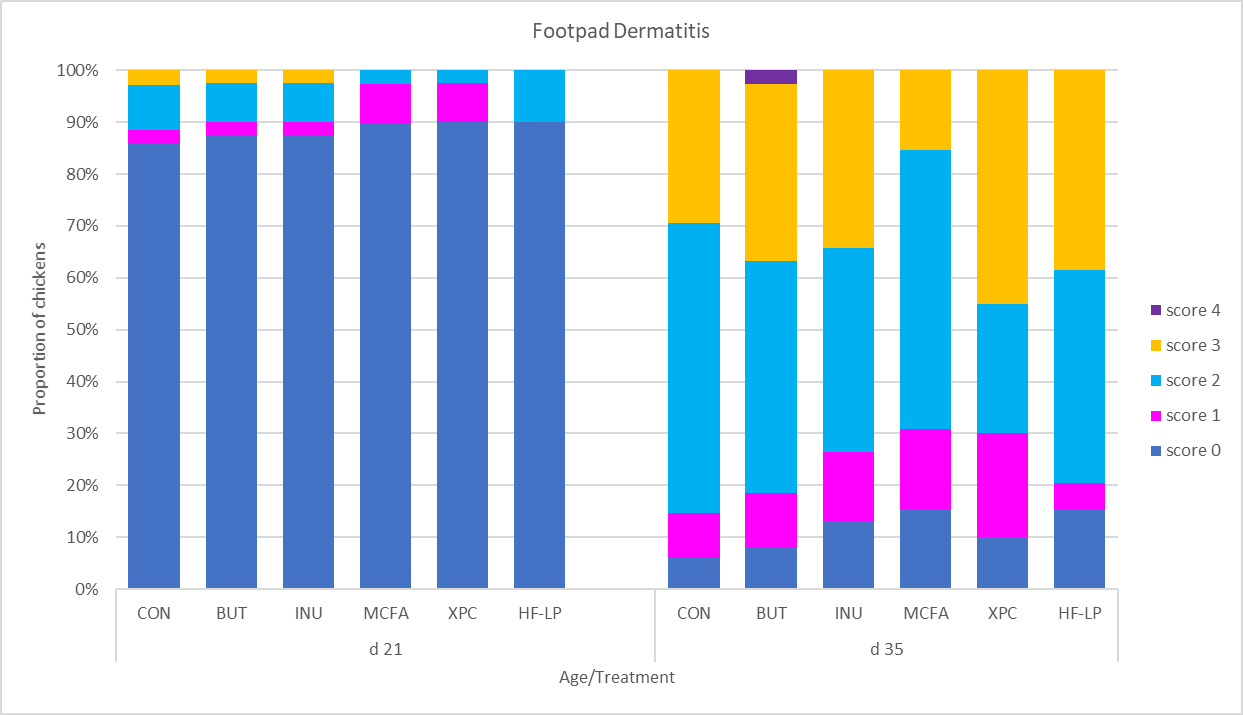


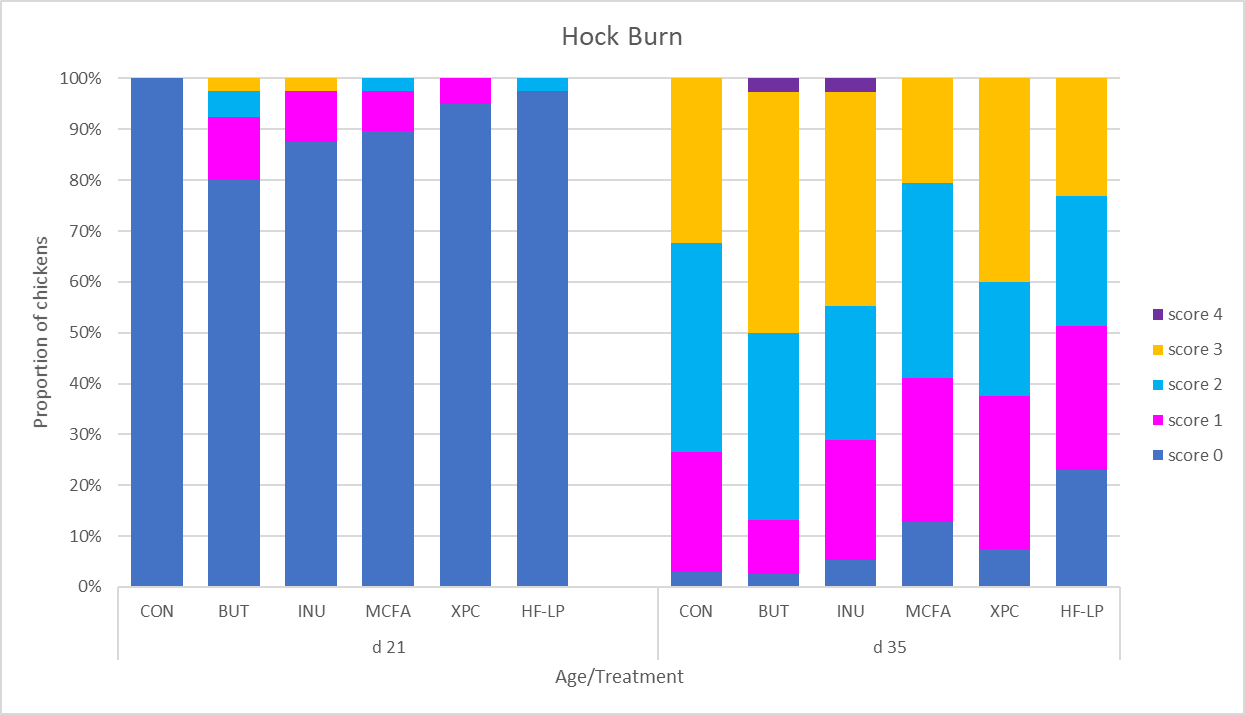

Supplement: Supplementary file 5 [file mmc5.docx]
