# Supplementary material for: Dietary strategies can increase cloacal endotoxin levels and modulate the resident microbiota in broiler chickens
Source: Poult Sci. 2023 Nov 20;103(2):103312. doi: 10.1016/j.psj.2023.103312 (PMC10762469; doi:10.1016/j.psj.2023.103312)
Supplement: Supplementary file 6 [file mmc6.docx]

**Supplementary File S6.** Estimates of linear regression slope of litter dry matter in week 1 to 4, and litter dry matter at d35. CON, control; BUT, butyrate; INU, inulin; MCFA, medium-chain fatty acids; XPC, Diamond XPC; HF-LP, high fiber-low protein; SED, Standard Error of Differences and P-value for treatment effects.

|  | **CON** | **BUT** | **INU** | **MCFA** | **XPC** | **HF-LP** | **SED** | ***P-value*** |
| --- | --- | --- | --- | --- | --- | --- | --- | --- |
| Slope | -14.08 | -14.13 | -13.65 | -13.26 | -13.76 | -13.52 | 0.3567 | **<0.001** |
| Dry matter Week 5 | 34.76 | 32.85 | 35.69 | 37.87 | 34.68 | 35.82 | 1.258 | **0.013** |
